# Catalytic Promiscuity Underpins Metabolic Resistance to Auxinic Herbicides in *Echinochloa phyllopogon*

**DOI:** 10.1021/acs.jafc.6c02002

**Published:** 2026-06-16

**Authors:** Pattarasuda Chayapakdee, Kowit Hengphasatporn, Takuya Yamaguchi, Danrui Su, Niña Gracel Dimaano, Yasuteru Shigeta, Yukari Sunohara, Hiroshi Matsumoto, Satoshi Iwakami

**Affiliations:** † Center of Excellence in Microbial Diversity and Sustainable Utilization, Agrobiodiversity in Highland and Sustainable Utilization Research Group, and Department of Biology, Faculty of Science, 26682 Chiang Mai University, Chiang Mai 50200, Thailand; ‡ Center for Computational Sciences, 13121 University of Tsukuba, Tsukuba 305-8573, Japan; § Biotechnology Research Center and Department of Biotechnology, Toyama Prefectural University, Toyama 939-0398, Japan; ∥ United Graduate School of Agricultural Science, 13125 Tokyo University of Agriculture and Technology, Fuchu 183-8509, Japan; ⊥ Institute of Weed Science, Entomology and Plant Pathology, College of Agriculture and Food Science, University of the Philippines Los Baños, Laguna 4031, Philippines; # Graduate School of Life and Environmental Sciences, University of Tsukuba, Tsukuba 305-8573, Japan; ∇ Institute of Agriculture, Tokyo University of Agriculture and Technology, Fuchu 183-8509, Japan

**Keywords:** CYP81A, evolution, metabolic resistance, molecular dynamics simulation, nontarget-site resistance, weed

## Abstract

Auxinic herbicides remain essential for weed control, yet metabolic resistance to these herbicides is poorly understood. Here we show that catalytically promiscuous CYP81A P450s from *Echinochloa phyllopogon* detoxify auxinic herbicides. *Arabidopsis thaliana* expressing CYP81A12 or CYP81A21 showed decreased sensitivity to quinclorac. In an *Escherichia coli* whole-cell assay, these enzymes produced a putative hydroxylated quinclorac metabolite. Docking and 300 ns molecular dynamics simulations indicated that active CYP81As maintain quinclorac in a productive pose close to the heme, with pocket geometry and water exclusion enabling precise substrate–heme alignment, whereas the inactive CYP81A18 retains quinclorac farther from the catalytic center. Consistent with resistant *E. phyllopogon*, transgenic *Arabidopsis* expressing CYP81A12 or CYP81A21 produced less ethylene after quinclorac treatment, linking detoxification to suppressed auxinic phytotoxic responses. CYP81As also conferred resistance to florpyrauxifen-benzyl and 2,4-D, highlighting cross-resistance across auxinic chemistries. These findings reveal a structural basis for CYP81A-mediated catalytic promiscuity underlying metabolic resistance to auxinic herbicides.

## Graphical abstract

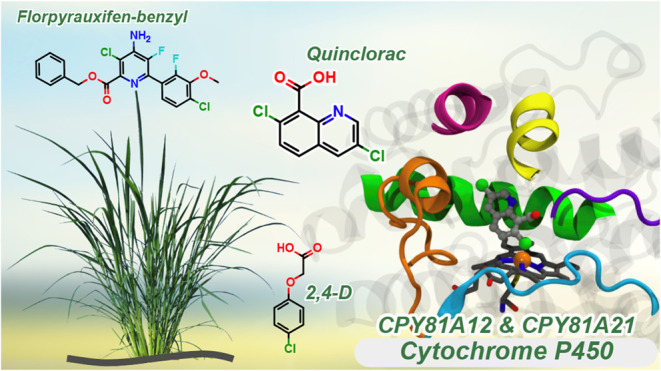

## Introduction

Weed control using herbicides has become an indispensable tool in modern crop production. However, continuous herbicide application imposes strong selection pressure, leading to the evolution of herbicide-resistant weed populations.^1^ Synthetic auxins, introduced as the first herbicide in 1945, have been widely used for decades and have remained effective with relatively low frequencies of resistance.^2^ Thus, while resistance to other herbicides has caused devastating problems, auxinic herbicides have continued to serve as valuable options for managing resistant weed populations. However, the recent deployment of genetically modified crops resistant to auxinic herbicides, along with the development of new selective auxinic herbicides for major crops, has resulted in a rapid increase in reports of resistant weeds.^3^ Addressing these resistant weeds requires a detailed understanding of their resistance mechanisms. Given the complex mode of action of auxinic herbicides, which mimic the plant hormone auxin, the molecular basis of resistance remains largely elusive. Unraveling these mechanisms is not only essential for sustainable weed management but also for advancing our understanding of auxin signaling and herbicide action.

As with other herbicide classes, resistance to auxinic herbicides can be classified into target-site resistance (TSR) and nontarget-site resistance (NTSR).^3^ While recent studies have begun to elucidate TSR mechanism for auxinic herbicides,^4–6^ NTSR mechanisms remain poorly understood. Proposed mechanisms of NTSR include reduced translocation, enhanced metabolic detoxification (*e*.*g*., by cytochrome P450 monooxygenases), and the activation of damage protection enzymes such as β-cyanoalanine synthase (β-CAS).^7^ However, the specific molecular players involved in auxinic herbicide NTSR are still largely unknown, with only a few candidates such as CYP72A recently proposed.^8^

Quinclorac, a widely used auxinic herbicide, strongly induces ethylene biosynthesis in plants, leading to the accumulation of hydrogen cyanide as a byproduct.^9^ Because hydrogen cyanide is phytotoxic, its excessive accumulation following quinclorac treatment is considered a primary cause of plant death.^10^ Accordingly, enhanced detoxification of hydrogen cyanide via β*-*CAS has been proposed as a resistance mechanism.^11^

Previously, we investigated quinclorac resistance in a multiple-herbicide resistant (MHR) population of the paddy weed *Echinochloa phyllopogon* collected in California, USA in 1997. Interestingly, this population exhibited resistance to quinclorac despite no known history of exposure to auxinic herbicides.^12^ Our earlier studies suggested that resistance to several nonauxinic herbicides in this population is conferred by a genetically inferred but as yet unidentified factor that drives the coordinated overexpression of multiple cytochrome P450 genes, *i*.*e*., *CYP81A12*, *CYP81A21*, and *CYP709C69*.^13^ In contrast, initial physiological analyses suggested that quinclorac resistance in this population could involve (1) increased β*-*CAS activity and (2) reduced ethylene production following quinclorac treatment.^12^ Subsequent analyses using recombinant inbred lines (RILs) derived from a sensitive (S) and a MHR line, however, showed that the increased β*-*CAS activity genetically segregated from the quinclorac resistance phenotype,^14^ whereas quinclorac resistance remained strongly associated with reduced ethylene production and with resistance to herbicides with different modes of action. These findings raised the possibility that the reduced ethylene production may result from rapid detoxification of quinclorac via overexpressed P450 enzymes.

In this study, we hypothesized that the catalytically promiscuous cytochrome P450s, CYP81A12 and CYP81A21, which are overexpressed in MHR *E. phyllopogon*, contribute to quinclorac resistance through metabolic detoxification. Using heterologous expression systems (*Arabidopsis thaliana* and *Escherichia coli*), *in vitro* metabolite analysis, and *in silico* structural modeling, we evaluated the quinclorac-metabolizing activity of these enzymes. These findings provide a mechanistic explanation for auxinic herbicide resistance and highlight how CYP81A-mediated metabolism can enable resistance across diverse herbicide chemistries.

## Materials and Methods

### Plant Material

Two inbred lines of *E. phyllopogon* were used: MHR line (line 511) and S line (line 401). These MHR and S lines were originally collected in 1997 from rice fields in California’s Sacramento Valley and were self-pollinated for three consecutive generations.^15^ The MHR line exhibits resistance to acetolactate synthase inhibitors,^16^ acetyl-CoA carboxylase inhibitors,^17^ a 1-deoxy-D-xylulose 5-phosphate synthase inhibitor,^18^ very-long-chain fatty acid elongase inhibitors,^19^ 4-hydroxyphenylpyruvate dioxygenase,^20^ in addition to auxinic herbicides shown in this study.

Transgenic *A. thaliana* plants overexpressing each gene from the *CYP81A* subfamily were generated previously.^21,22^ Herbicide sensitivity of each line was described previously.^20^ 81A12#21 and 81A21#6 were used unless stated otherwise.

### Herbicide Sensitivity Assays

Unless otherwise stated, herbicide sensitivity of *A. thaliana* was evaluated as described previously.^22^ Briefly, sterile seeds were transferred to Murashige and Skoog (MS) medium plates containing herbicides and kept in a growth chamber at 22 °C under constant light (40–90 μmol m^–2^s^–1^) for up to 30 d. For the experiment shown in Figures 1A and S1, 5-day-old seedlings germinated on quinclorac-free MS medium were transferred to MS medium containing quinclorac.

**Figure 1:**
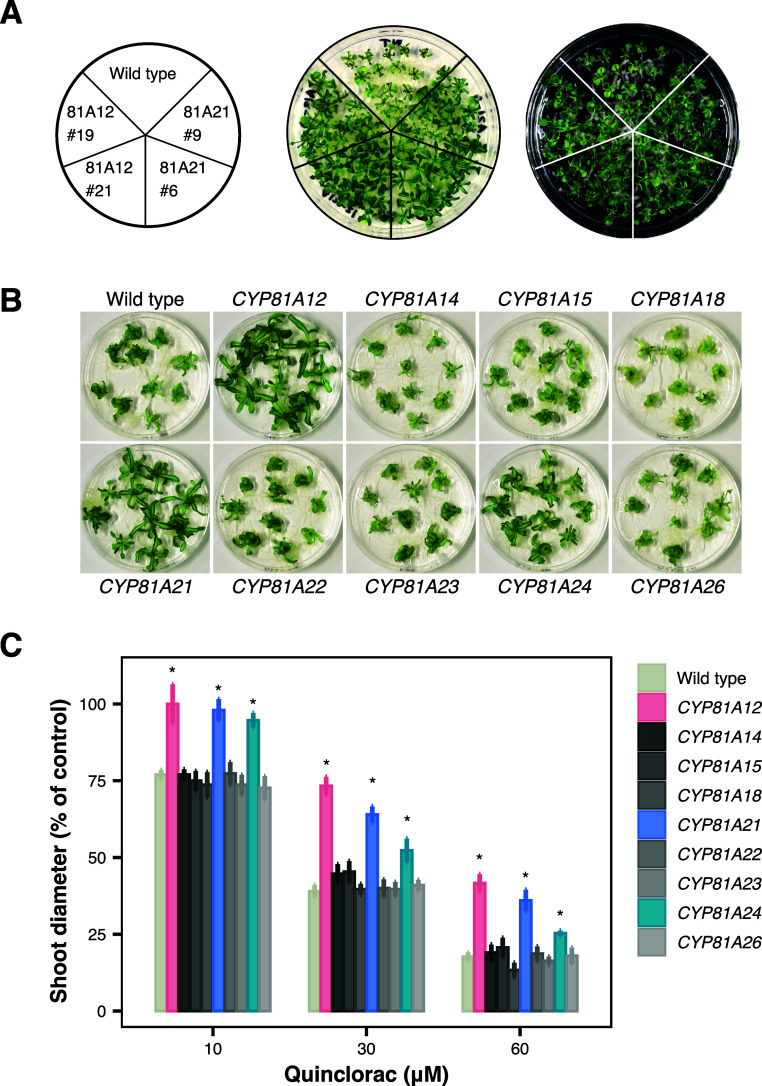
Quinclorac responses of *A. thaliana* expressing CYP81A P450s of *E. phyllopogon*. (A) *A. thaliana* phenotypic response to 100 μM quinclorac. Seeds were germinated on MS medium for 5 d and subsequently transferred to quinclorac-containing medium for an additional 15 d. (B) *A. thaliana* phenotypic response to 30 μM quinclorac after 30 d of cultivation. (C) Quantification of growth response. Bar graph shows mean ± SD of three biological replicates. * Significantly different from the wild type (*P* < 0.05, Dunnett’s test).

Herbicide sensitivity assays for the S and MHR lines of *E. phyllopogon* were conducted using solid medium culture. Seeds from the S and MHR lines were sterilized with 2.5% (w/v) sodium hypochlorite for 15 min, further sterilized with 0.2% (w/v) sodium hypochlorite for 30 min and washed three times with sterile water. The seeds were germinated on wet filter paper in Petri dishes for 3 d in a growth chamber set at 25 °C with a 12 h photoperiod (250 −300 μmol m^–2^ s^–1^). The seedlings were then transferred to MS solid medium containing herbicides.

For foliar spray treatment of florpyrauxifen-benzyl, germinated *E. phyllopogon* seeds were transplanted to soil with four plants per pot and cultivated under LED with 13 h photoperiod and 27 ± °C. Plants under 3.8 to 4-leaf-stage were sprayed according to Iwakami et al.^23^ Florpyrauxifen-benzyl (Loyant 2.7% EC; Corteva Japan Ltd., Tokyo, Japan) were treated at the 1/27X, 1/9X, 1/3X, 1X, 3X, and 9X of the recommended rate (50 g a.i. ha^–1^) with 0.05% detergent (Surfactant 30; Maruwa Biochemical, Tokyo, Japan). Malathion (Marathon 50%; Sumitomo Chemical Co., Ltd., Tokyo, Japan) was foliar-applied at 300 g a.i. ha^–1^, 3.5 h prior to herbicide treatment. Plants were collected at 16 d after application and dried for 3 d at 80 °C for dry weight measurement.

### Quantification of Ethylene Production

Ethylene production was determined according to the method described previously^14^ with some modifications. *A. thaliana* plants were germinated in solid MS medium under the same conditions as above for 30 d. Then, the roots of grown plants were immersed in quinclorac solution for 12 h and three plants were transferred to a 30 mL glass vial containing filter paper and 2 mL distilled water. The vial was sealed tightly and maintained under germination conditions. After 96 h incubation, 1 mL gas samples were withdrawn by syringe from the vial and quantified for ethylene by gas chromatography (GC-17A; Shimadzu Corp., Kyoto, Japan). The GC contained a glass column packed with Unipak S (GL Sciences Inc., Tokyo, Japan) and a hydrogen flame ionization detector. The temperatures of the chromatograph oven, injection, and detector were 60, 120, and 120 °C, respectively. The flow rate of the nitrogen carrier gas and the pressures of hydrogen (fuel gas) and compressed air (oxidant gas) were adjusted to 50 mL min^–1^, 60 kPa, and 50 kPa, respectively. The quinclorac concentrations applied to the plants were 0, 300, or 1000 μM. Ethylene production was measured in triplicate.

### Quinclorac Metabolism

Details of *E. coli* expressing each *CYP81A* gene from *E. phyllopogon* was described in our previous study.^20^ Briefly, the *E. coli* C41­(DE3) lines carry pET28 vector with a *CYP81A* gene, pCDF vector with *ATR2* and 5-aminolevurinic acid synthase (*HemA*) genes, and pGro7 with chaperonin gene. *E. coli* colonies were inoculated into LB containing glucose (1% w/v), kanamycin (50 μg mL^–1^), streptomycin (50 μg mL^–1^), and chloramphenicol (34 μg mL^–1^), and cultured at 37 °C overnight. A 10 μL aliquot was transferred to 1 mL of autoinduction medium, followed by culture at 26 °C for 24 h. Quinclorac (0.2 mM) and arabinose (2 μg mL^–1^) were added, and the culture was continued at 30 °C for 24 h.

Samples were analyzed using liquid chromatography-tandem mass spectrometry (LC-MS/MS) with a COSMOSIL 2.5C18-MS-II column under the following conditions: mobile phase A, 0.1% formic acid in water; mobile phase B, acetonitrile; and a 10–60% linear gradient of B for 8 min and 60% B for 1 min at a flow rate of 0.4 mL min^–1^. Mass spectrometry was conducted in positive ion mode using an LCMS-8030 (Shimadzu, Japan), with multiple reaction monitoring (MRM) used to detect hydroxylated quinclorac metabolite under the following conditions: positive-ion mode with MRM transition of *m*/*z* 258 [M + H]+ to *m*/*z* 177. As authentic hydroxylated quinclorac metabolites are not commercially available, MRM transitions for the hydroxylated metabolites were set based on the MS/MS fragmentation patterns of quinclorac.

### *In Silico* Analyses

#### CYP81As Protein Structure Modeling

The protein sequences of CYP81As were derived from previously identified nucleotide sequences.^21^ The 3D structural models were generated using integrated template-based and *de novo* prediction techniques via the Robetta server.^24^ Hydrogen atoms were then added to the protein models, and heme groups were positioned within the active sites through structural superimposition with a protein template (PDB code: 6VBY).^25^ Structural minimization of all models was performed using the ff19SB AMBER force field^26^ for standard amino acids, while the penta-coordinate ferric high-spin (heme) was minimized using parameters from previous research implemented in AmberTools 21.^27^

#### Molecular Docking

To predict the possible conformation and binding affinity between quinclorac and CYP81As, the minimized structures of CYP81As served as templates for molecular docking using Autodock Vina 1.1.2.^28^ The structure of quinclorac was constructed using GaussView 6.0 and optimized with Gaussian 16 using the DFT B3LYP/6–31G* basis set.^29^ Ligand and protein structures were charged and converted to PDBQT format using AutoDockTools.^30^ The binding site in CYP81As was defined around the heme, surrounded by SRS1 to SRS6, with grid dimensions of 15 Å × 15 Å × 15 Å, defined by the AutoGrid 4.0.0 module.^30^ The ligand-binding pose and interactions were analyzed in 2D and 3D using Discovery Studio Visualizer Software^31^ and Chimera USCF.^32^ The quinclorac/CYP81As complexes with the lowest binding energies were selected for molecular dynamics (MD) simulations to assess the stability of ligand binding and explore molecular mechanisms.

#### Molecular Dynamics Simulation of Quinclorac/CYP81As Complexes

The initial structures of the quinclorac/CYP81As complexes were subjected to MD simulations using the AMBER20 software package.^33^ Each complex was first prepared by examining its protonation state using the PDB 2PQR web tools.^34^ Hydrogen atoms and ions were then added to each system, and hydrogen atoms were minimized using the SANDER tool from AmberTools 21.^33^ Each complex was solvated with a TIP3P explicit water model, maintaining a cutoff distance of 12 Å from the protein’s surface. This was followed by 3000 steps of steepest descent and 3000 steps of conjugate gradient minimization. The systems were gradually heated to 300 K over 20 ps under periodic boundary conditions with a canonical ensemble (NVT). The simulations were then continued at 300 K for 300 ns under an isothermal–isobaric ensemble (NPT). The stability of the systems was examined by monitoring the root-mean-square deviation (RMSD) and the displacement between iron in the heme and quinclorac (*d*_Fe‑Quinclorac_) over the 300 ns simulation calculated by the CPPTRAJ module implemented in AmberTools 21. The binding pocket volume of CYP81A enzymes was calculated using POVME3.0(1) with default parameters. The grid was centered 2 Å above the heme group, which corresponds to the enzymatic active site. A spherical radius of 15 Å was used to define the pocket region. For each enzyme, 30 representative structures were extracted from the 300 ns MD trajectory in PDB format at 10 ns intervals. These structures were used to capture the dynamic changes in pocket volume during the simulation. The two-dimensional free energy landscape (2D-FEL) profile was performed based on the Markov state models (MSMs) using PyEMMA. From the last 50 ns of each system, a total of 5000 snapshots were extracted and used for per-residue decomposition free energy calculations using the molecular mechanics generalized Born solvent-accessible surface area (MM/GBSA) method,^35^ which identifies key amino acids involved in ligand binding to the protein.

### Bioinformatics

Previously conducted RNA-seq data of the MHR and S lines (accession number DRA013092)^13^ was reanalyzed using the reference genome of the MHR line (https://plantgarden.jp/). Raw reads were quality-filtered and adapter-trimmed using fastp (v0.22.0)^36^ with a minimum quality score of Q30 and minimum read length of 80 bp. Subsequently, clean reads were aligned to the reference genome using STAR (v2.7.10b)^37^ with “--sjdbOverhang 99”. Read counts were quantified at the gene level using featureCounts (v2.0.6). Differential expression analysis was performed by edgeR package (v4.8.2) (FDR < 0.05, fold change ≥ 3).^38^

The Aux/IAA gene family was identified by DIAMOND BLAST^39^ through a comparative analysis of the complete amino acid sequences of rice (https://rapdb.dna.naro.go.jp/) and *E. phyllopogon*. Polymorphisms were detected as described previously.^40^ Briefly, clean reads (accession number PRJDB14855) were mapped with Bowtie2,^41^ and sequence variants were called using BCFtools^42^ and VCFtools.^43^

The CDS sequences were used to construct the phylogenetic tree. Multiple sequence alignment of protein sequences was performed using MAFFT (v7.525).^44^ Codon-based alignment was then generated using PAL2NAL (v14)^45^ by mapping the protein alignment back to the corresponding CDS sequences. Maximum likelihood phylogenetic tree was constructed using IQ-TREE (v3.0.1)^46^ with the best-fit substitution model selected by ModelFinder based on the Bayesian information criterion, and branch support was assessed using 1000 bootstrap replicates.

### Statistical Analysis

Statistical analyses were performed using R.^47^ For multiple comparisons, Dunnett’s test was used, and for two-group comparisons, Welch’s *t*-test was applied. Dose responses curves were drawn using drc package (v. 3.0–1).^48^

## Results

### Quinclorac Response of *A. thaliana* Expressing *CYP81A12/21*

Quinclorac resistance of RILs derived from a S and a MHR line of *E. phyllopogon* linked to the resistance to most of the herbicides, which is explained by coregulation of multiple herbicide-metabolizing P450,^14^ *i*.*e*., catalytically promiscuous CYP81A12 and CYP81A21, and highly substrate-specific CYP709C69. Although no activity to quinclorac was confirmed for CYP709C69,^13^ CYP81A12 and CYP81A21 had not been analyzed yet. To confirm the role of these two P450s in quinclorac resistance, we tested the sensitivity of the previously established *A. thaliana* lines expressing *CYP81A12* and *CYP81A21*. In our previous research, the lines exhibited marked resistance responses to many herbicides by 10 d cultivation on herbicide-supplemented MS media.^20^ However, in the case of quinclorac response, plant response was not very clear at 10 d (Figure S1). Considering that *A. thaliana* is naturally tolerant to quinclorac, we further continued cultivation of the plants. Interestingly, the longer the plants were cultivated, the clearer the growth differences became. *A. thaliana* plants expressing these P450s grew better in quinclorac-containing medium compared to wild-type plants at 15 d (Figure 1A).

Therefore, *A. thaliana* lines with higher expression, i.e., 81A12#21 and 81A21#6,^21^ were further investigated under prolonged cultivation. The shoot diameters of the transformants were significantly larger under the 10, 30 and 60 μM treatments at 30 d (Figure 1B,C).

To further confirm the role of CYP81As in plant response to quinclorac, other *CYP81A* genes present in *E. phyllopogon*, *i*.*e*., *CYP81A14*/*15*/*18*/*22*/*23*/*24*/*26*, which are not associated with resistance in the MHR line, were also investigated. The results revealed that, in addition to *CYP81A12* and *CYP81A21*, *CYP81A24* also conferred mild resistance to quinclorac in *A. thaliana* (Figure 1B,C). These findings indicate that overexpression of specific CYP81As in plants can confer resistance to quinclorac.

### Role of CYP81As in Quinclorac Metabolism

To determine whether CYP81As metabolize quinclorac, we performed an *E. coli* whole-cell metabolism assay using a previously established protocol. Cultures were supplemented with 0.2 mM quinclorac and incubated for 24 h, followed by LC-MS/MS analysis of the culture medium. A peak corresponding to hydroxylated quinclorac was monitored in MRM mode. Because authentic hydroxylated quinclorac metabolites are not commercially available, the MRM transitions were determined based on the MS/MS fragmentation pattern of quinclorac. The protonated molecular ion [M + H]^+^ of quinclorac was detected at *m*/*z* 242 (Figure 2A). MS/MS fragmentation of this ion produced three product ions at *m*/*z* 224, 194, and 161 (Figure 2B). The fragment ion at *m*/*z* 224 is attributed to the loss of H_2_O (18 Da) from the molecular ion, and further dissociation via the loss of CO (28 Da) and sequential loss of CO (28 Da) and Cl (35 Da) yields fragment ions at *m*/*z* 196 and 161, respectively (Figure 2C).^49^ Among these fragments, the ion at *m*/*z* 161 was the most abundant. As hydroxylation (+O) increases the molecular mass by 16 Da, the MRM transition for the hydroxylated quinclorac was set to *m*/*z* 258 (242 + 16) → 177 (161 + 16).

**Figure 2:**
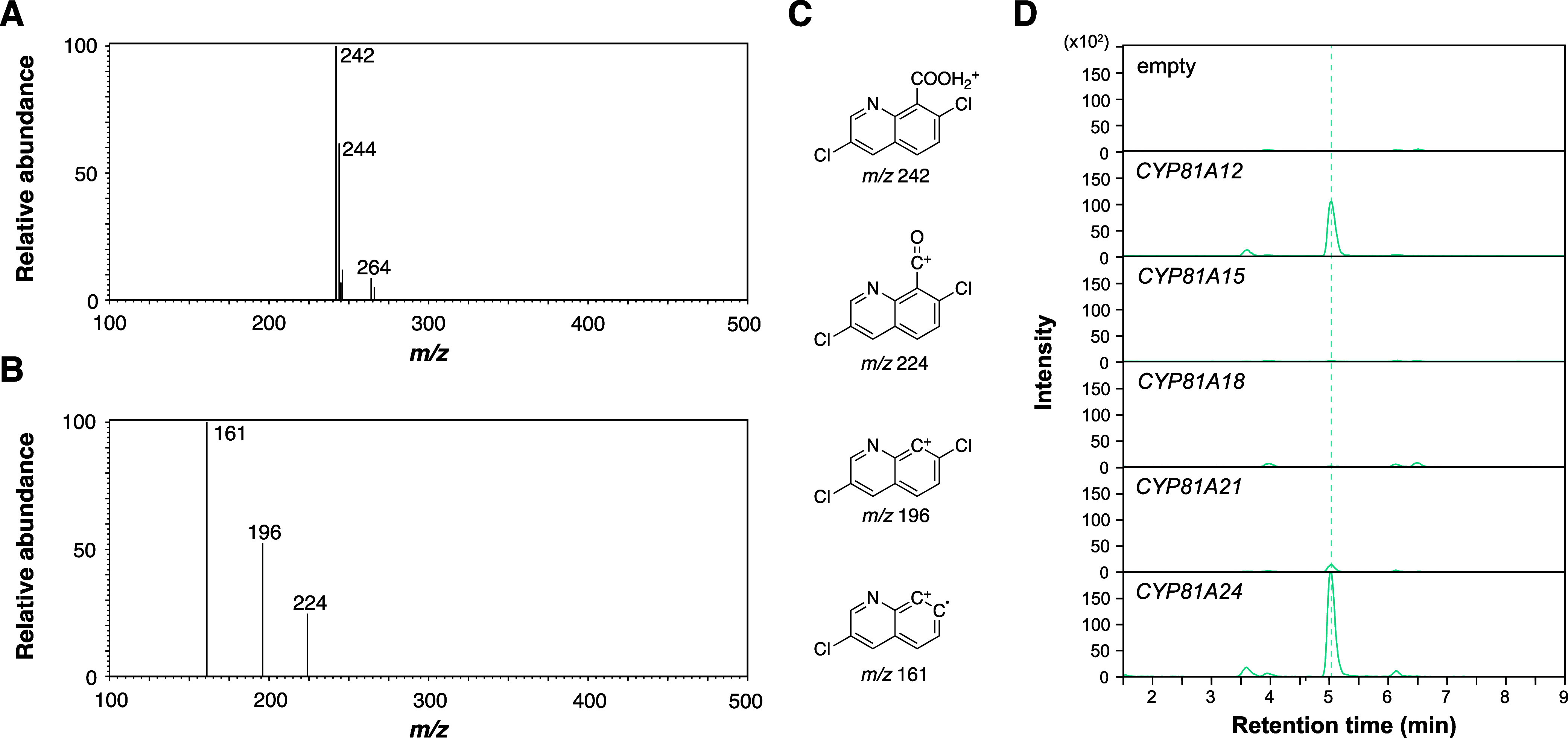
Detection of putative hydroxylated quinclorac in *CYP81A-*expressing*E. coli* (A) Mass spectrum of quinclorac. (B) MS/MS spectrum of quinclorac ion at *m*/*z* 242. (C) Fragmentation pathway of quinclorac.^9^ (D) *E. coli* harboring empty vector (pET28a­(+)) and CYP81As were cultured in the medium containing quinclorac. Its metabolites were analyzed using liquid chromatography-tandem mass spectrometry with multi reaction monitoring *m*/*z* 258 [M + H]^+^ → *m*/*z* 177.

We tested three CYP81A enzymes that conferred quinclorac resistance in *A. thaliana* and two others, CYP81A15 and CYP81A18, which did not confer resistance. A peak corresponding to hydroxylated quinclorac was monitored. A clear peak with a retention time of 5.1 min was identified in the culture solution of *E.* *coli* with *CYP81A12*, *CYP81A21*, and *CYP81A24* (Figure 2), in accordance with the results in *A. thaliana*. The findings indicate that CYP81A enzymes have hydroxylation activity toward quinclorac.

### Quinclorac/CYP81As Interactions via *In Silico* Study

To investigate the quinclorac-metabolizing activity of CYP81As in *E. phyllopogon*, we conducted an *in silico* analysis of their interactions with quinclorac. Due to the absence of available crystal structures, the 3D structures of the CYP81As were generated using the Robetta server. The geometry and stereochemistry of these models were validated using Ramachandran plots (Figure S2). The structural models revealed the presence of six putative substrate recognition sites (SRSs), highlighting the high degree of similarity between CYP81A12, CYP81A21, and CYP81A24, with approximately 75% sequence identity. In contrast, CYP81A18, which does not confer quinclorac resistance, shares only 50% sequence identity (Figures S3, S4).

The molecular docking results demonstrated that quinclorac could bind to the active sites of the minimized structures of CYP81As located near the heme catalytic region. Consistent with the *A. thaliana* and *E. coli* studies, the binding energy scores for quinclorac interacting with CYP81A12 (−7.6 kcal mol^–1^), CYP81A21 (−7.3 kcal mol^–1^), and CYP81A24 (−7.9 kcal mol^–1^) were more favorable compared with CYP81A18 (−6.6 kcal mol^–1^), which is the negative control in this study (Figure S5). These interactions are influenced by the hydrophobicity, hydrophilicity, and amphipathicity of the SRSs, which determine how quinclorac accesses to and is stabilized in the binding pocket. Several residues within the SRS domains interacted with the core structure of quinclorac through van der Waals (vdW), π–π, and π-alkyl interactions, consistent with previous studies in other P450s.^50,51^ In CYP81A12 and CYP81A21, polar residues facilitated interactions with the carboxyl group of quinclorac through hydrogen bonds or ionic interactions, guiding it toward the heme. CYP81A24, with the highest amphipathicity (8.00%), exhibited a balance of hydrophobic and hydrophilic residues, allowing dynamic binding and stabilizing both aromatic and polar moieties in quinclorac. In contrast, CYP81A18, with the highest hydrophobicity (54.67%), interacted primarily with quinclorac through vdW forces, which likely restricted its movement deeper into the pocket and hindered access to the heme, potentially affecting catalytic activity.

All-atom MD simulations were conducted to further investigate the molecular basis of metabolism. The binding stability and conformational dynamics of the quinclorac–CYP81A complexes were assessed over 300 ns trajectories (Movie 1). The root-mean-square deviation (RMSD) analysis of quinclorac and the backbone of CYP81As indicated high stability throughout the simulations (Figure 3A). Whereas, in the CYP81A21 and CYP81A24 systems, quinclorac exhibited slight fluctuations due to the rotational freedom of its carboxyl group. Analysis of the distance between the center of mass of quinclorac and the heme iron (*d*_Fe‑Quinclorac_) throughout the MD trajectory suggested that quinclorac gradually moved closer to the catalytic site of the heme in CYP81A12 (∼5 Å), CYP81A21 (∼7 Å), and CYP81A24 (∼6 Å), whereas it remained more distant in CYP81A18 (∼11 Å) (Figure S5B and Movie 1). The hydration dynamics around heme in the pocket were analyzed across four systems by evaluating the total number of water molecules (#Water) within the inner (0–3.4 Å) and outer (3.4–5 Å) hydration shells of Fe-heme. CYP81A12 and CYP81A21 showed low water occupancy at the Fe-heme, indicating a less solvent-exposed or more hydrophobic environment. CYP81A24 exhibited moderate water occupancy with slight fluctuations, suggesting periodic changes in solvent accessibility. Due to the motion of quinclorac, we found that water can access the pocket of CYP81A18 and move close to the Fe-heme, which is higher than the other systems, leading to the loose binding of quinclorac in the binding pocket (Figure S5C).

**Figure 3:**
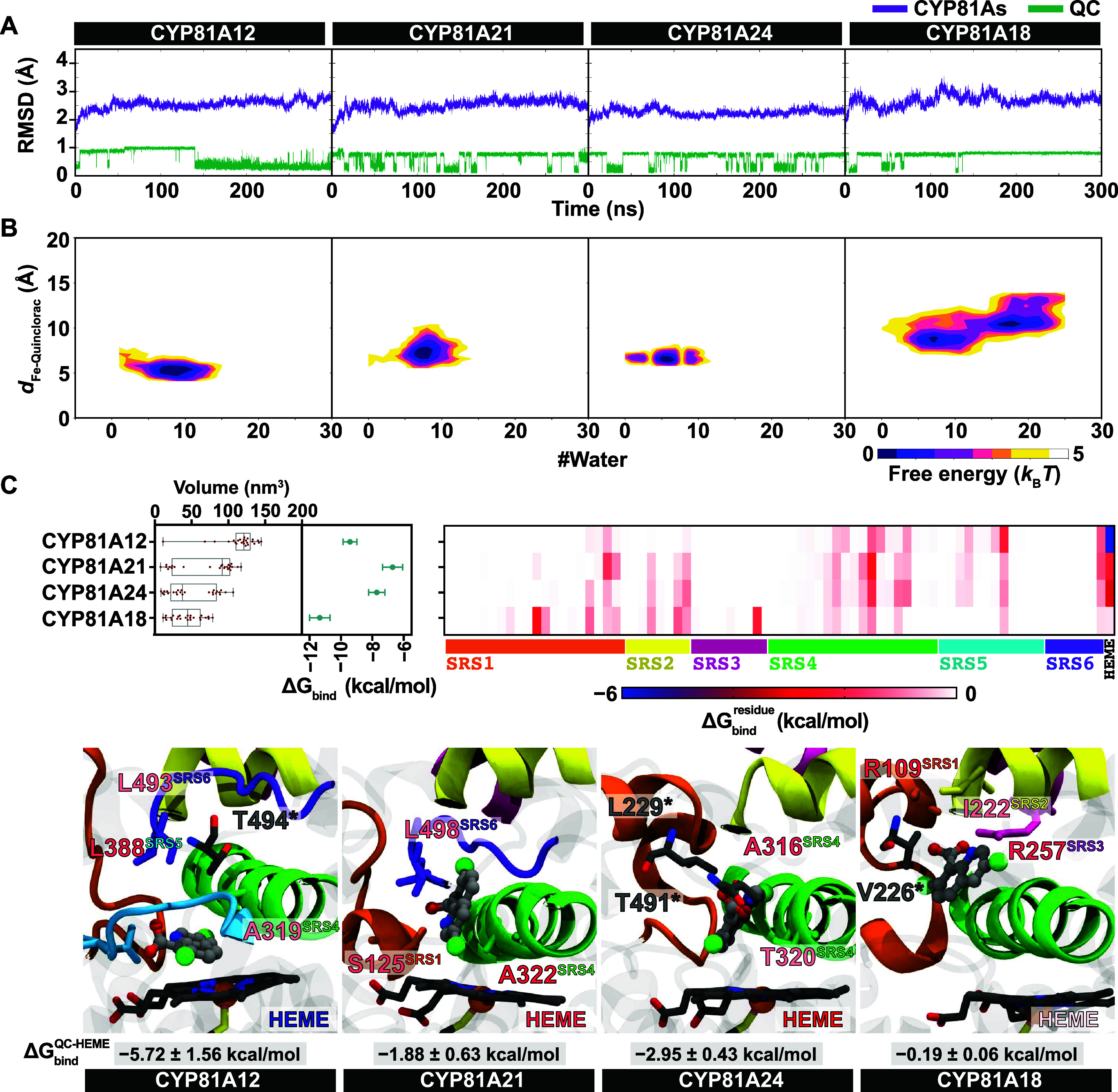
Structural dynamics and binding profiles of quinclorac in CYP81A12, CYP81A21, CYP81A24, and CYP81A18 variants. (A) Cα-RMSD and quinclorac–heme distance (*d*_Fe–Quinclorac_) over 300 ns MD simulations. (B) Two-dimensional free energy landscapes (2D-FEL) showing *d*_Fe–Quinclorac_ versus the number of water molecules in the binding pocket (#Water). (C) Binding pocket volume, binding free energy (Δ*G*_bind_), and per-residue energy decomposition (Δ*G*_bind_^residue^QUOTE) calculated by the MM/GBSA method, along with representative binding poses.

The CYP81A18 system revealed a unique two-dimensional free energy landscape (2D-FEL) pattern characterized by two global minima across a broad chemical space. These minima were observed at both lower and higher water densities within the binding pocket compared to the other systems (Figure 3B). In contrast, the other three systems displayed a narrower 2D-FEL with a single global minimum, suggesting higher ligand stability. During the MD simulation, CYP81A12 exhibited the largest binding pocket volume (average from the last 50 ns: 130.56 ± 9.54 nm^3^) and the highest range of structural fluctuations, supporting its superior metabolic activity (Figures 3C and S5). The larger, more flexible pocket likely enhances substrate binding and positioning, improving catalytic efficiency for quinclorac metabolism. On the other hand, CYP81A18 had a smaller, narrower binding pocket (average from the last 50 ns: 48.82 ± 21.17 nm^3^), which likely restricted substrate access to the heme and hindered proper positioning, resulting in reduced activity.

The binding (Δ*G*_bind_QUOTE) and decomposition free energy (Δ*G*_bind_^residue^QUOTE) calculations, derived from the final 50 ns of MD trajectory, reveal that quinclorac binds more effectively to the SRSs of CYP81A18 (−11.35 ± 0.65 kcal mol^–1^) than CYP81A12 (−9.42 ± 0.44 kcal mol^–1^), CYP81A21 (−6.70 ± 0.64 kcal mol^–1^), and CYP81A24 (−7.72 ± 0.52 kcal mol^–1^). However, in CYP81A18, quinclorac is located far from the catalytic center (Figure 3C). Consistent with the interaction profile from Δ*G*_bind_^residue^QUOTE quinclorac in CYP81A18, it moves away from the heme and interacts stably with R257 on SRS3. In CYP81A21 and CYP81A24, quinclorac forms stable π-alkyl interactions with residues A322 and A316 in SRS4 (Figure 3C). Based on the interaction profile (Figure 3C), quinclorac consistently interacts with the α-helix in the SRS4 and leucine in SRS6 in all systems. The close proximity of these interactions to the heme group in CYP81A12, CYP81A21, and CYP81A24 indicates stronger binding affinities and greater potential for quinclorac metabolism. This could explain why these CYP81As are more efficient at quinclorac metabolism, as their interaction networks are better aligned with the catalytic core. The interaction shift observed in CYP81A18, involving regions distant from the heme, may underlie its reduced capacity for quinclorac metabolism, reinforcing the specificity of certain CYP81A proteins in quinclorac resistance. These findings suggest that the spatial arrangement of SRSs in relation to the heme group is critical for the efficiency of herbicide metabolism, offering valuable insights into the molecular mechanisms underlying resistance.

### Association of Quinclorac Resistance and Ethylene Production

In our previous study, the complete association between quinclorac resistance and reduced ethylene production was observed in the RILs of *E. phyllopogon*.^14^ Together with the above findings that CYP81A12 and CYP81A21 metabolize quinclorac, we hypothesized that reduced ethylene biosynthesis in resistant *E. phyllopogon* is caused by the decreased quinclorac level due to faster quinclorac metabolism. To test this hypothesis, ethylene production in *A. thaliana* lines expressing *CYP81A12*/21 was measured after quinclorac application. Here, *CYP81A24* line was excluded since the gene is not involved in quinclorac resistance in *E. phyllopogon*.

*A. thaliana* plants grown on MS medium for 30 d were transferred to quinclorac solutions (0, 300, and 1000 μM) for 12 h, and then kept in glass vials (Figure 4A). After 96 h, ethylene levels were measured using gas chromatography. The results demonstrated that *A. thaliana* carrying *CYP81A12*/21 produced significantly less ethylene (578 and 892 nl gFW^–1^, respectively) than wild-type plants (2791 nl gFW^–1^) (Figure 4B). This study confirmed that upregulation of *CYP81A12*/21 enhances quinclorac inactivation and reduces ethylene production in plants.

**Figure 4:**
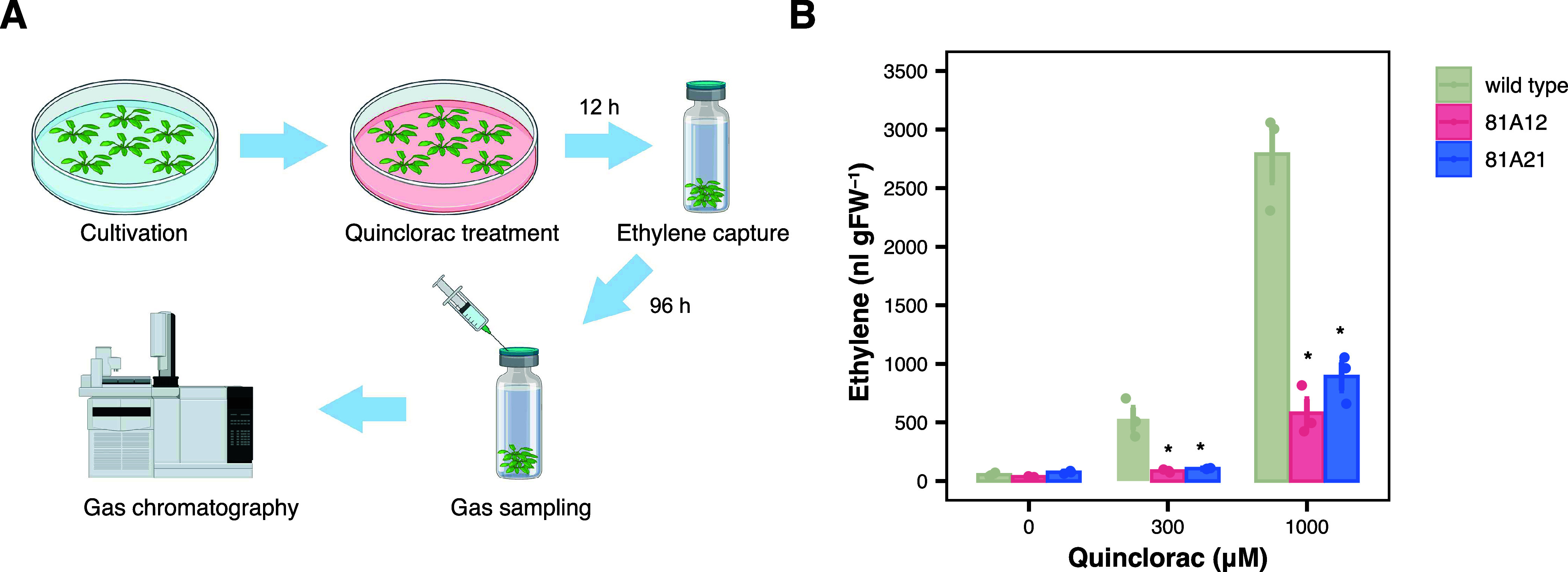
Ethylene production after quinclorac treatment in *A. thaliana*. (A) Methodology of ethylene quantification. Created with BioRender.com. (B) Ethylene levels in quinclorac treated plants. Data showed mean ± SD of triplicates (3 vials, 3 plants/vial). * Significantly different from group wild type (*P* < 0.05, Dunnett’s test).

### Cross-Resistance to Auxin Herbicides by CYP81A P450s

Having identified the metabolic activity of CYP81A toward the quinolinecarboxylate herbicide quinclorac, we next evaluated its activity against other auxinic herbicides belonging to chemical classes distinct from quinclorac (Figure 5A). We tested the arylpicolinate herbicide florpyrauxifen-benzyl, a recently commercialized auxinic herbicide used for the control of *Echinochloa* spp. in rice cultivation. We also examined the phenoxycarboxylate herbicide 2,4-D, the earliest auxinic herbicide and still widely used today. Because 2,4-D has little herbicidal activity against grasses, including *Echinochloa* spp., at recommended field application rates, treatments were conducted at high concentrations using the pure compound.

**Figure 5:**
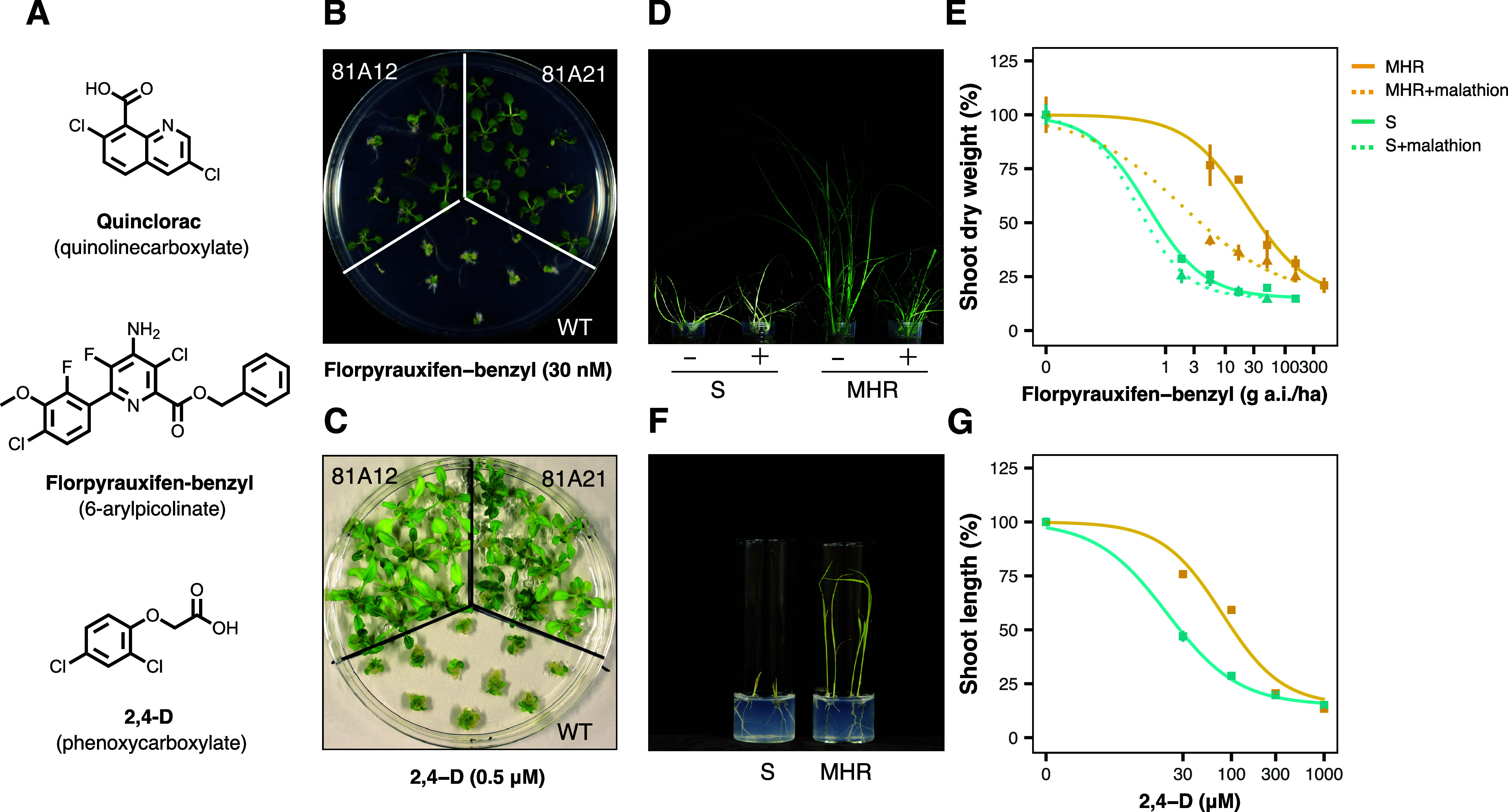
Role of CYP81A P450s in plant sensitivities to other auxinic herbicides. (A) Chemical structure of quinclorac, florpyrauxifen-benzyl and 2,4-D. (B) Florpyrauxifen-benzyl responses of *A. thaliana* expressing *CYP81A12* and *CYP81A21*. (C) 2,4-D responses of *A. thaliana* expressing *CYP81A12* and *CYP81A21*. (D, E) Florpyrauxifen-benzyl responses of sensitive (S) and multiple-herbicide-resistant (MHR) *E.* *phyllopogon*. −, no-malathion; +, malathion. Bars, ± SE (*n* = 3). (F, G) 2,4-D responses of sensitive and MHR *E. phyllopogon*. Bars, ± SE (*n* = 9).

*A. thaliana* lines expressing the quinclorac-metabolizing genes *CYP81A12* and *CYP81A21* exhibited resistance to both florpyrauxifen-benzyl and 2,4-D (Figure 5B,C). Consistent with this, 2,4-D resistance in these lines was also reported in a previous study.^52^ Based on these results, we expected that the MHR line overexpressing CYP81A12 and CYP81A21 would also exhibit resistance to these herbicides. As expected, the MHR line showed clear resistance to florpyrauxifen-benzyl and 2,4-D (Figure 5D–G).

To verify that resistance to florpyrauxifen-benzyl is mediated by CYP81A12 and CYP81A21, we confirmed that florpyrauxifen-benzyl resistance was partially reversed by pretreatment with the P450 inhibitor malathion (Figure 5D,E). Furthermore, using RILs, we demonstrated that resistance to florpyrauxifen-benzyl did not segregate from resistance to the acetolactate synthase inhibitor bensulfuron-methyl (Figure S7). In this MHR line, resistance to these herbicides has been repeatedly shown to cosegregate with resistance to other herbicides, including quinclorac.^14,17,21,22^ Together, these results indicate that CYP81A P450s are capable of detoxifying auxinic herbicides with diverse chemical structures, consistent with their previously reported ability to metabolize herbicides with differing modes of action.

### Analysis of Target-Site Mutations

Finally, we examined target-site mutations known to confer resistance to auxinic herbicides. In weeds, resistance to auxinic herbicides has been associated with mutations in the degron or degron tail regions of the Aux/IAA gene family.^4–6^ A near telomere-to-telomere genome assembly is available for the MHR line, in which a total of 52 Aux/IAA genes were identified. RNA-seq analysis did not detect any differentially expressed genes within the Aux/IAA family. In addition, nonsynonymous polymorphisms were identified using previously reported resequencing data from susceptible lines (Eph1.1ch1B.g014490, A5E and A272V; Eph1.1ch3B.g025580, Y155H; Eph1.1ch4A.g041370, G123R; Eph1.1ch4B.g006790, T49A; first residue, MHR line). However, these substitutions were not located in the degron or degron tail regions and were therefore considered to represent natural variation rather than resistance-associated mutations.

## Discussion

Although resistance to auxinic herbicides has recently emerged in several weed species, the underlying molecular mechanisms remain poorly understood, with only a few cases involving TSR. Quinclorac has long been considered to undergo limited metabolic degradation in plants,^10^ and its resistance mechanisms have therefore received little attention. Previous physiological studies on an MHR line of *E. phyllopogon* proposed mechanisms such as reduced ethylene production and increased β-CAS activity;^12^ however, our earlier work showed that β-CAS overexpression is not genetically associated with quinclorac resistance in this line.^14^ In this study, heterologous expression of CYP81A12 and CYP81A21 in *A. thaliana* and *E. coli* revealed that CYP81A-mediated quinclorac metabolism can account for the observed reduction in ethylene production. The relatively low level of quinclorac resistance conferred by CYP81A12 and CYP81A21 in *A. thaliana* is consistent with the modest resistance phenotype observed in the MHR line of *E. phyllopogon*. This may explain why quinclorac metabolism has been underestimated to date, as even minor differences in metabolic capacity were not previously recognized as having measurable effects on herbicide responses in plants. Taken together, our findings provide strong evidence that cytochrome P450–mediated metabolism plays a key role in quinclorac resistance in *E. phyllopogon*.

CYP81A12 and CYP81A21 likely represent the primary determinants of quinclorac sensitivity, although they may not fully account for all variation. Several lines of evidence strongly support this conclusion: resistance to nearly all herbicides tested cosegregates in the progeny, CYP81A12 and CYP81A21 are consistently overexpressed in the MHR line,^13,17,21^ the association of resistance level between *E. phyllopogon* ^14^ and CYP81A-expressing *A. thaliana*­(Figure 1), and treatment with malathion partially reverses quinclorac resistance.^12^ Furthermore, the likelihood of TSR is low, as no auxinic herbicides effective against *Echinochloa* spp. had been used in California prior to the collection of this line, and no previously reported TSR mutations were detected in Aux/IAA genes. Notably, overexpression of *CYP81A12* and *CYP81A21* is thought to be controlled by a single regulatory factor, and several genes are coregulated with these P450s, including *CYP709C69*, which metabolizes diclofop-methyl.^13^ Such coordinated regulation suggests that additional factors influencing auxinic herbicide metabolism or sensitivity cannot be excluded. Co-regulation of multiple detoxification genes has recently emerged as a novel mechanism underlying herbicide resistance, particularly in the context of multiple-herbicide resistance,^53^ and further studies will be required to elucidate the regulatory network governing this coordinated response.

In agricultural weeds, catalytically promiscuous P450s were first identified through studies of this MHR *E. phyllopogon*.^17,21,22^ Later, the detoxification capabilities of CYP81As have been extensively evaluated, revealing that CYP81A12, CYP81A21, and CYP81A24 can metabolize a wide range of herbicides used in modern agriculture.^20^ This study further revealed that the promiscuity of CYP81A P450s extends to auxinic herbicides 2,4-D and florpyrauxifen-benzyl. Notably, florpyrauxifen-benzyl, a 6-arylpicolinate synthetic auxin, was first commercialized in 2018—nearly two decades after the collection of the MHR *E. phyllopogon* in California. This temporal gap highlights that broad-spectrum metabolic resistance can predate the deployment of new herbicide chemistries, posing a serious challenge to proactive resistance management. CYP81A24 had already been shown to metabolize herbicides targeting six distinct modes of action—acetolactate synthase, phytoene desaturase, 4-hydroxyphenylpyruvate dioxygenase, 1-deoxy-D-xylulose 5-phosphate synthase, acetyl-CoA carboxylase, and protoporphyrinogen oxidase.^17,54^ It is now evident that CYP81A24 also metabolizes herbicides targeting a seventh mode of action, synthetic auxin.

Meanwhile, the underlying reasons why these P450s can detoxify such a diverse array of herbicides remained unclear. In this study, *in silico* analyses were conducted to investigate the metabolic mechanism of quinclorac. By comparing CYP81A enzymes capable of metabolizing quinclorac (CYP81A12/21/24) with those unable to do so (CYP81A18), we explored the structural and functional characteristics of CYP81A enzymes. The docking studies and molecular dynamics simulations corroborated the experimental findings, showing that CYP81A12, CYP81A21, and CYP81A24 have stronger binding affinities for quinclorac than CYP81A18. The water molecules near Fe in CYP81A18 (Figure S5) may obstruct substrate access and reduce the conformational flexibility of quinclorac, limiting its catalytic efficiency compared to the others. Therefore, the solvent environment could be crucial for the activity of CYP81A (Figures 3C, S5C, and S6). The pocket volume analysis showed that quinclorac bound to CYP81A12 has the largest pocket, allowing greater flexibility and easier access to the heme center. In contrast, CYP81A18 has the smallest pocket and strongest binding to surrounding residues, restricting quinclorac’s movement toward the heme and reducing metabolic activity due to interactions with SRS1, SRS2, and SRS4 (Figure 3C). The larger pocket and lower water content in CYP81A12, CYP81A21, and CYP81A24 likely enhance their metabolic performance. The detailed analysis of SRSs and the binding free energy profiles sheds light on the molecular mechanisms that underlie quinclorac resistance, particularly the key residues that facilitate effective herbicide metabolism. Nevertheless, the precise mode of quinclorac metabolism by CYP81As, *e*.*g*., the specific hydroxylation site, remains unclear. Several high computational approaches, *i*.*e*., quantum mechanics/molecular mechanics (QM/MM) calculation or cluster model analysis, can be further utilized to confirm the reaction of CYP81As-quinclorac at the quantum level. Furthermore, it is essential to evaluate the detoxification mechanisms for other herbicides. While plant P450 enzymes were long thought to exhibit high substrate specificity, recent discoveries have identified P450s, including CYP81A and others, with broad substrate versatility.^55^ Computational simulations hold promise for uncovering the structural and functional characteristics that enable these P450s to process diverse substrates.

One of the significant insights revealed through this series of studies on quinclorac resistance in the MHR *E. phyllopogon* ^12,14^ is its contribution to understanding the mode of action of synthetic auxins. While the reception and signaling pathways of endogenous auxins are well understood, the mechanisms by which synthetic auxins exert their effects remain unclear.^56^ It is widely believed that ethylene production induced by synthetic auxin application plays a central role in the processes leading to plant death. Our analysis of *A. thaliana* expressing the quinclorac-metabolizing P450 revealed a negative correlation between ethylene release and synthetic auxin perception, which supports earlier studies highlighting the link between ethylene evolution and plant death.^11^ However, our research strongly suggests that hydrogen cyanide, a byproduct of ethylene biosynthesis previously considered a key factor in plant death, is not involved in this process.^14^ Recent studies have also suggested the possibility that the accumulation of abscisic acid (ABA), independent of ethylene evolution, may contribute to plant death.^57^ Further investigation of ABA-related responses using our *E. phyllopogon* materials may provide additional insights into the mode of action of synthetic auxins.

Our research has progressively uncovered key molecular players involved in MHR in *E. phyllopogon*. In particular, we have identified the overexpression of promiscuous cytochrome P450 enzymes, as well as the coordinated upregulation of multiple detoxification-related enzymes.^13^ These findings have significantly advanced our ability to predict cross-resistance patterns associated with metabolism-based herbicide resistance.^20^ However, despite these advances, the regulatory mechanisms driving the simultaneous overexpression of these detoxification genes remain elusive. The strikingly similar expression patterns observed across independently evolved MHR populations suggest the involvement of common upstream regulatory factors.^13^ With the growing availability of genomic resources, including multiple genome assemblies^40,58^ and recombinant inbred lines (RILs),^21^ *E. phyllopogon* is emerging as a powerful model for studying MHR in weeds. Harnessing these resources will be essential for achieving a comprehensive understanding of metabolism-based herbicide resistance, a critical mechanism in modern weed management, and for informing the development of effective and sustainable control strategies.

## Supplementary Material





## Notes

Quinclorac responses of *Arabidopsis thaliana* expressing CYP81A12 and CYP81A21 of *Echinochloa phyllopogon*, Five-day-old plants were transferred to 100 μM quinclorac medium (Figure S1); Ramachandran plots of ψ versus φ angles for all amino acids in CYP81A12, CYP81A21, CYP81A24, and CYP81A18, generated using RAMPAGE in UCSF Chimera, Blue dots represent individual residues, while red and green areas indicate favored and allowed regions, respectively, reflecting overall protein structure quality (Figure S2); Structural models of CYP81A12, CYP81A21, CYP81A24, and CYP81A18 are shown as both superimposed and individual structures. Pairwise structural alignment of substrate recognition sites (SRSs) highlights domain identity, visualized as a grid map with color intensity ranging from white (0%) to red (100%) (Figure S3); Pairwise structural alignment of SRS domains among CYP81A12, CYP81A21, CYP81A24, and CYP81A18. Structural identity between substrate recognition sites (SRSs) is shown based on pairwise comparisons of each CYP81A variant (Figure S4); Structural and dynamic analysis of quinclorac binding in CYP81A variants. (A) SRS1–SRS6 sequence alignment, binding poses at 0 ns, and 2D interaction maps showing key residue contacts and Δ*G*_bind_ values, (B) Heatmap of quinclorac–heme distance (*d*_Fe–Quinclorac_) over 300 ns MD trajectory, (C) Time evolution of water molecules in the binding pocket (Figure S5); Volume analysis of CYP81A variant binding pockets over 300 ns MD simulations, The blue dots represent the volume of the CYP81A variant at each specific time point (Figure S6); Herbicide responses of F7 progeny lines (A) Florpyrauxifen-benzyl responses of bensulfuron-methyl-sensitive and -resistant lines, (B) Summary of sensitivity to florpyrauxifen-benzyl and bensulfuron-methyl in F7 progeny lines (Figure S7) (PDF)
The MD trajectory of quinclorac binding in CYP81A variants over 300 ns is shows together with the quinclorac–heme distance (*d*_Fe‑Quinclorac_) (MP4)
