# Supplementary material for: Catalytic Promiscuity Underpins Metabolic Resistance to Auxinic Herbicides in Echinochloa phyllopogon
Source: J Agric Food Chem. 2026 Jun 16;74(25):19454–64. doi: 10.1021/acs.jafc.6c02002 (PMC13329992; doi:10.1021/acs.jafc.6c02002)
Supplement: Supplementary file 1 [file jf6c02002_si_001.pdf]

Catalytic promiscuity underpins metabolic resistance to auxinic herbicides in *Echinochloa phyllopogon*

Pattarasuda Chayapakdee[a], Kowit Hengphasatporn[b*], Takuya Yamaguchi[c], Danrui Su[d], Niña Gracel Dimaano[e], Yasuteru Shigeta[b], Yukari Sunohara[f], Hiroshi Matsumoto[f], and Satoshi Iwakami[d,g*]

[a]Center of Excellence in Microbial Diversity and Sustainable Utilization, Agrobiodiversity in Highland and Sustainable Utilization Research Group, and Department of Biology, Faculty of Science, Chiang Mai University, Chiang Mai, 50200 Thailand.

[b]Center for Computational Sciences, University of Tsukuba, Tsukuba, 305-8573 Japan.

[c]Biotechnology Research Center and Department of Biotechnology, Toyama Prefectural University, Toyama, 939-0398 Japan.

[d]United Graduate School of Agricultural Science, Tokyo University of Agriculture and Technology, Fuchu, 183-8509 Japan.

[e]Institute of Weed Science, Entomology and Plant Pathology, College of Agriculture and Food Science, University of the Philippines Los Baños, Laguna 4031, Philippines.

[f]Graduate School of Life and Environmental Sciences, University of Tsukuba, Tsukuba, 305-8573 Japan.

[g]Institute of Agriculture, Tokyo University of Agriculture and Technology, Fuchu, 183-8509 Japan.

[*] Correspondence to: K Hengphasatporn (kowith@ccs.tsukuba.ac.jp), S Iwakami (iwakamis@go.tuat.ac.jp)

Supplementary Methods

Herbicide sensitivity assays in F7 lines

For florpyrauxifen-benzyl, Echinochloa phyllopogon plants were grown to the four-leaf stage and treated by foliar spraying, as described in the Materials and Methods. A detergent (Surfactant 30; Maruwa Biochemical) was added at a final concentration of 0.1%. Herbicide sensitivity was evaluated 9 days after treatment.

For bensulfuron-methyl, the assay was conducted according to Iwakami et al.[1] Briefly, germinated seeds were placed on MS medium, and herbicide sensitivity was evaluated after 7 days.

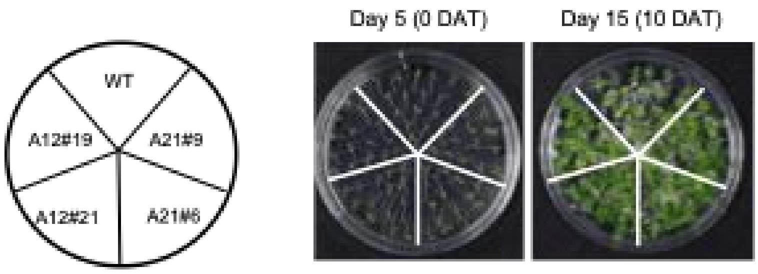

Figure S1. Quinclorac responses of *Arabidopsis thaliana* expressing *CYP81A12* and *CYP81A21* of *Echinochloa phyllopogon.*

Five-day-old plants were transferred to 100 μM quinclorac medium.

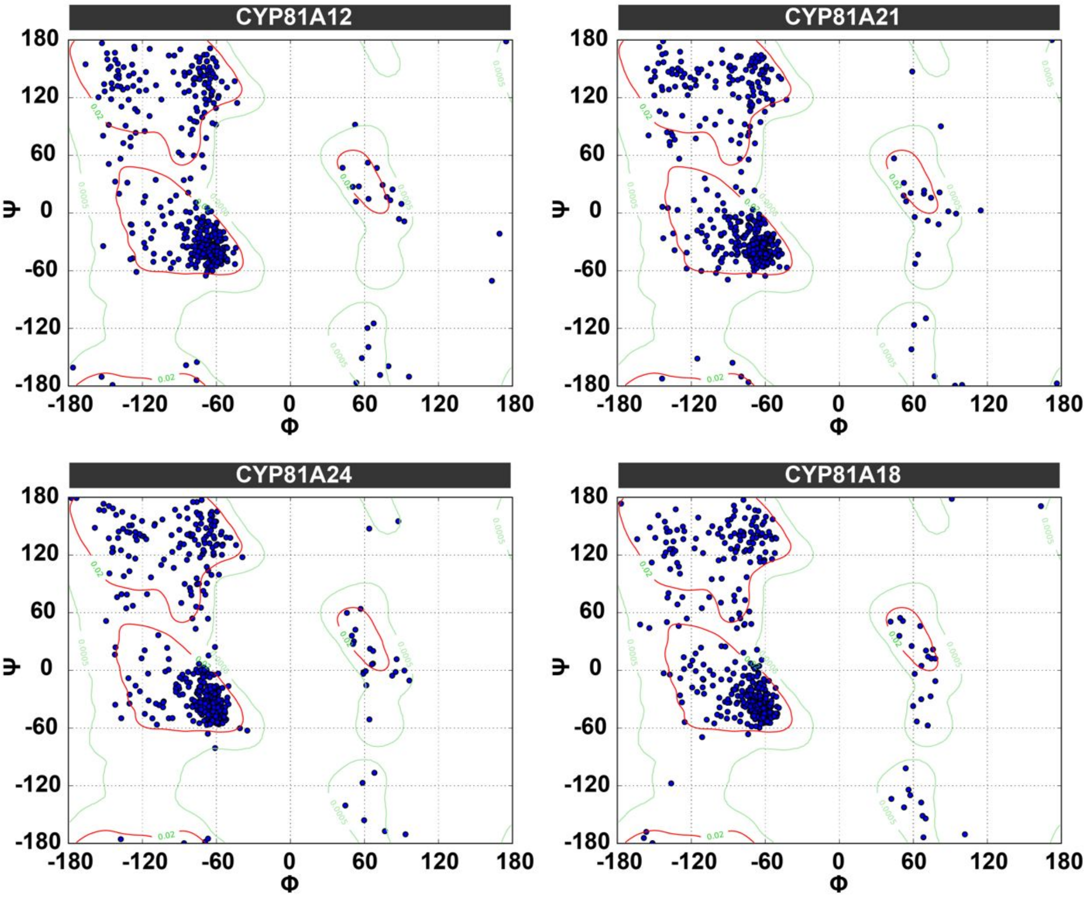

Figure S2. Ramachandran plots of ψ versus φ angles for all amino acids in CYP81A12, CYP81A21, CYP81A24, and CYP81A18, generated using RAMPAGE in UCSF Chimera. Blue dots represent individual residues, while red and green areas indicate favored and allowed regions, respectively, reflecting overall protein structure quality.

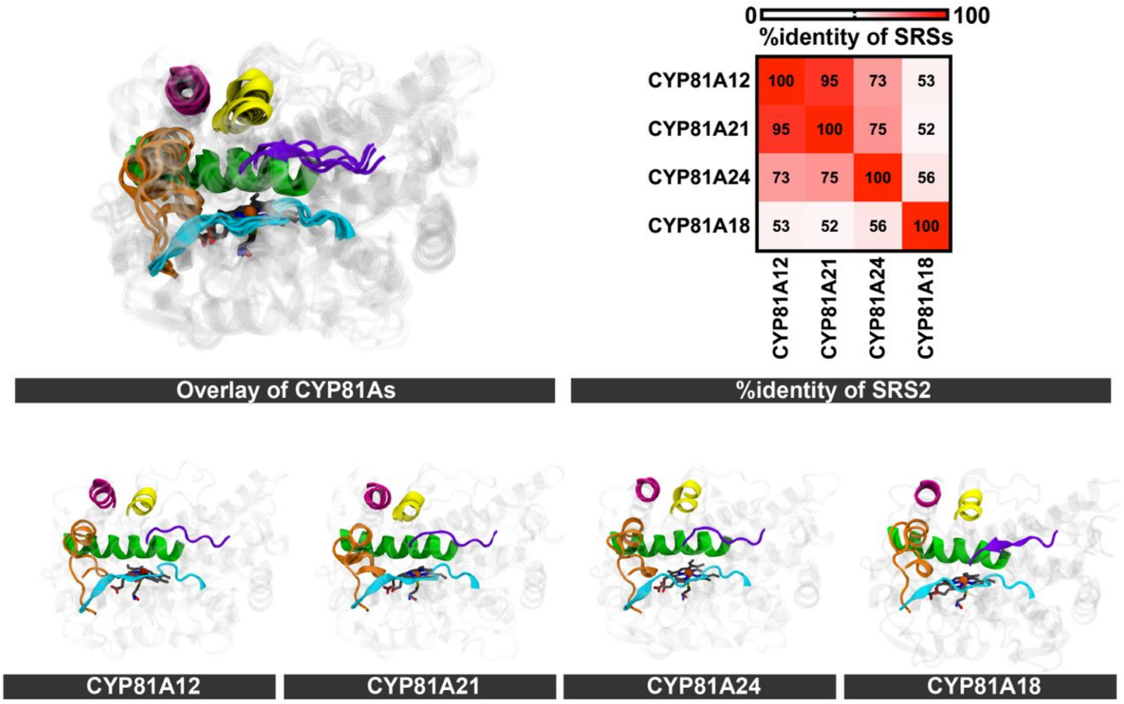

Figure S3. Structural models of CYP81A12, CYP81A21, CYP81A24, and CYP81A18 shown as both superimposed and individual structures. Pairwise structural alignment of substrate recognition sites (SRSs) highlights domain identity, visualized as a grid map with color intensity ranging from white (0%) to red (100%).

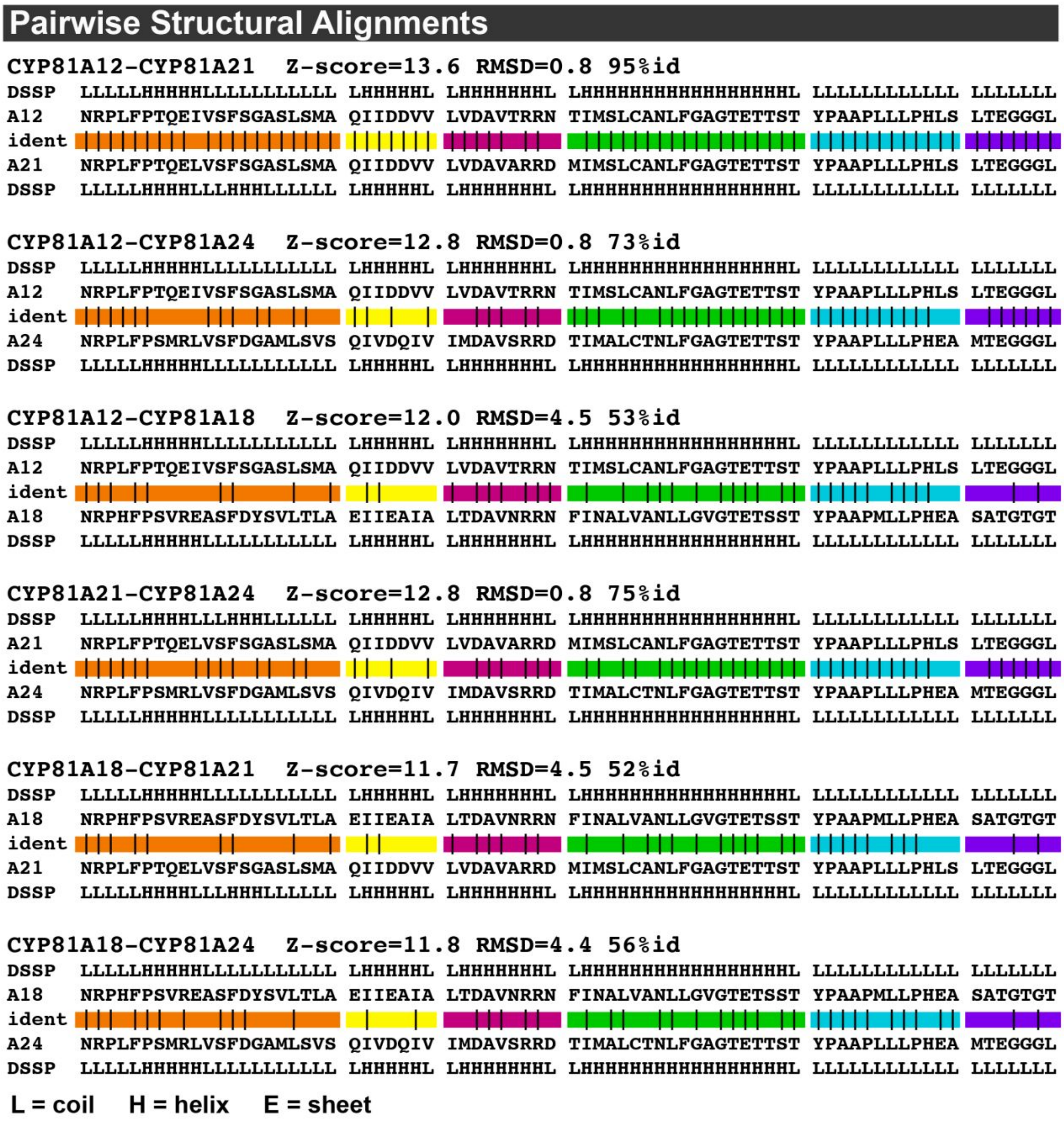

Figure S4. Pairwise structural alignment of SRS domains among CYP81A12, CYP81A21, CYP81A24, and CYP81A18. Structural identity between substrate recognition sites (SRSs) is shown based on pairwise comparisons of each CYP81A variant.

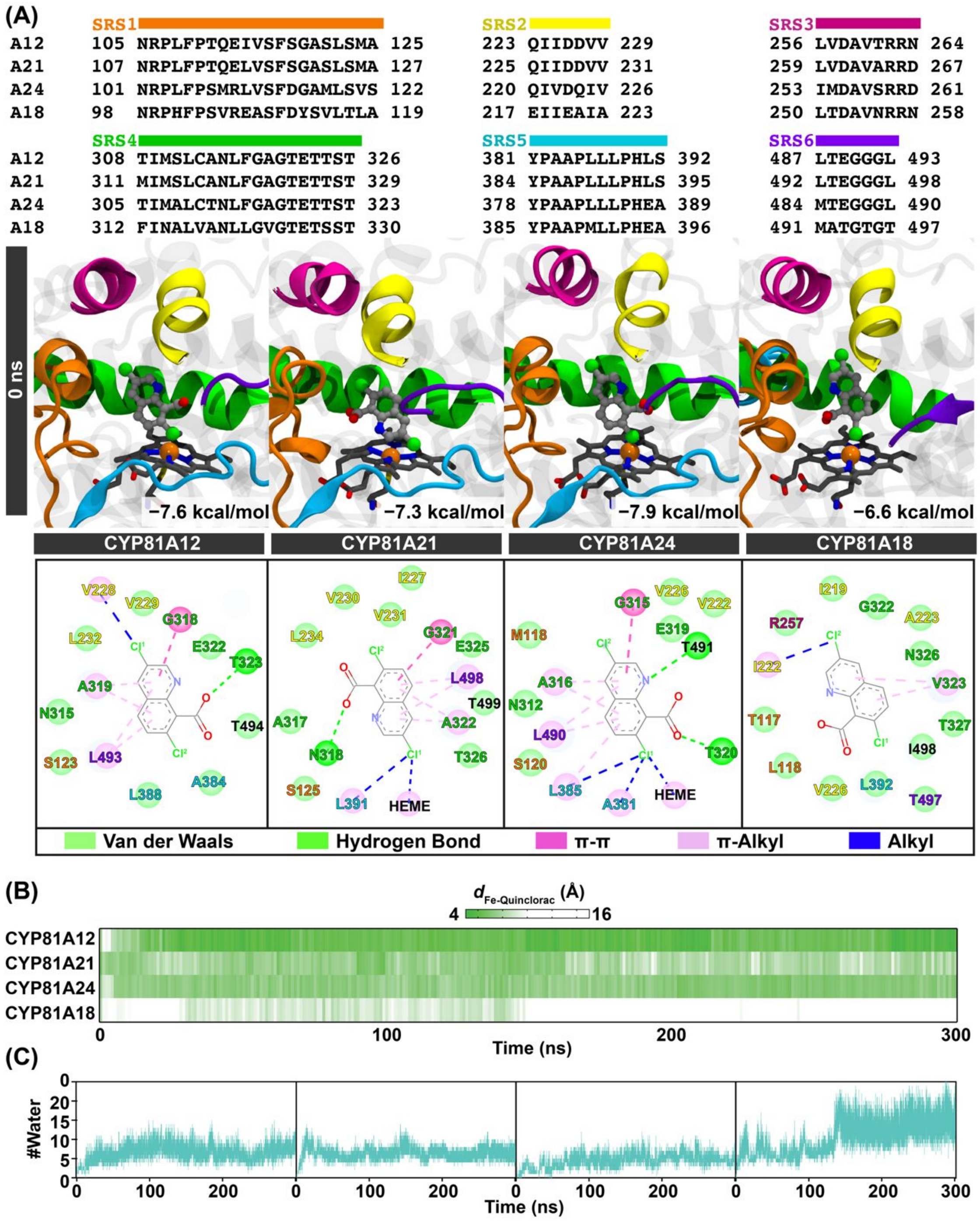

Figure S5. Structural and dynamic analysis of quinclorac binding in CYP81A variants.

(A) SRS1–SRS6 sequence alignment, binding poses at 0 ns, and 2D interaction maps showing key residue contacts and ΔG_{bind} values. (B) Heatmap of quinclorac–heme distance ($d_{Fe\text{–}Quinclorac}$) over 300 ns MD trajectory. (C) Time evolution of water molecules in the binding pocket.

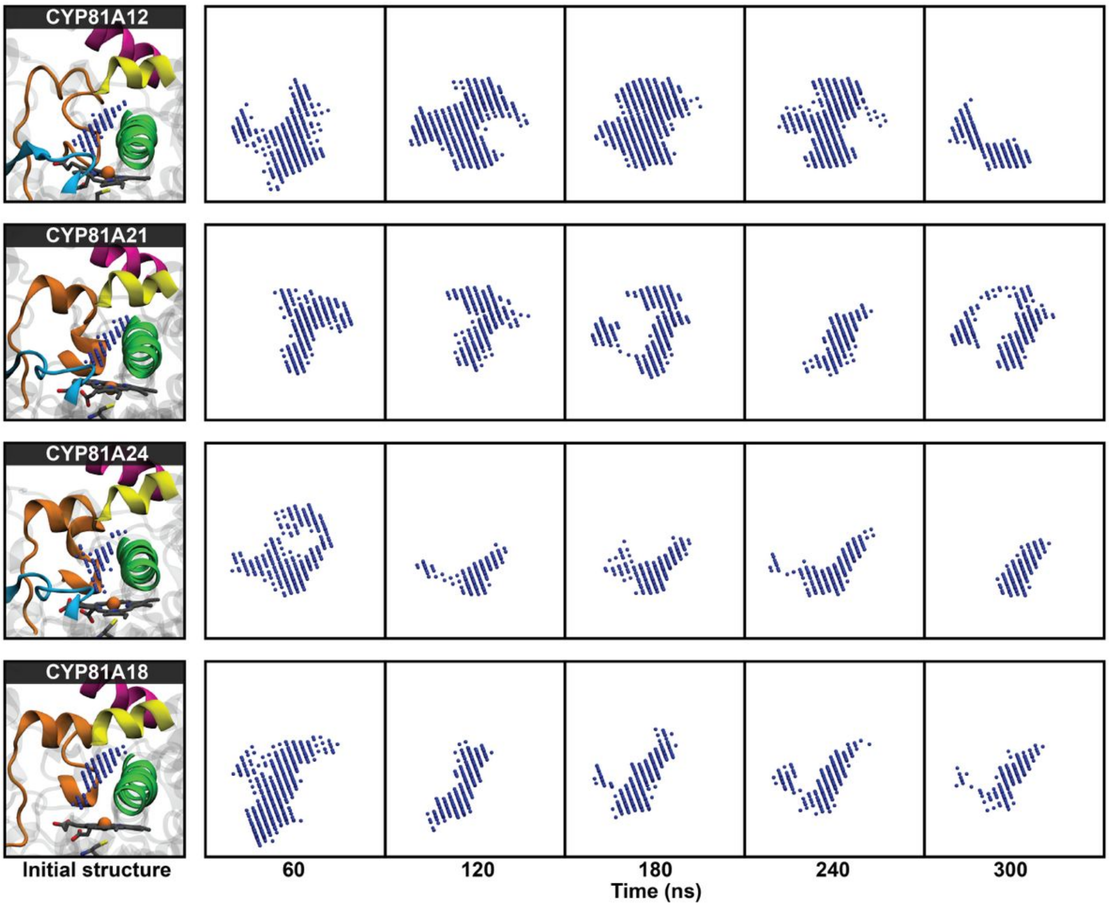

Figure S6. Volume analysis of CYP81A variant binding pockets over 300 ns MD simulations. The blue dots represent the volume of the CYP81A variant at each specific time point.

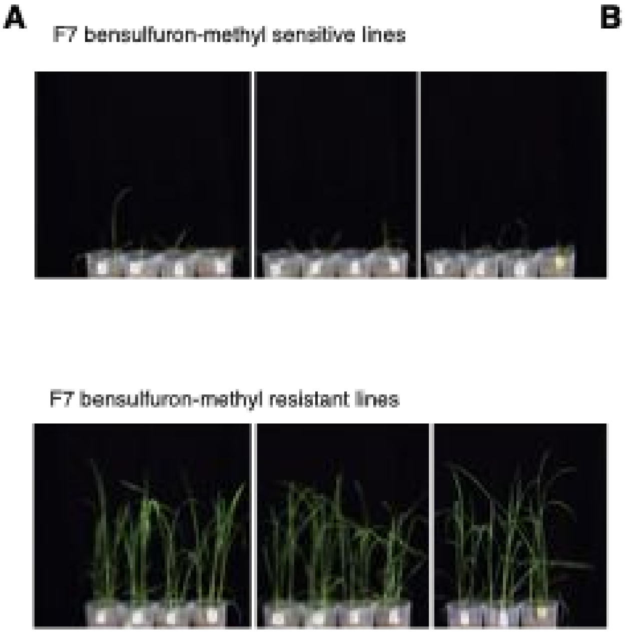

B

F7 Lines	Florpyrauxifen-benzyl	Bensulfuron-methyl
S	S	S
5	S	S
6	S	S
9	S	S
10	S	S
20	S	S
23	S	S
29	S	S
39	S	S
40	S	S
41	S	S
42	S	S
43	S	S
46	S	S
50	S	S
54	S	S
R	R	R
1	R	R
15	R	R
21	R	R
22	R	R
24	R	R
31	R	R
32	R	R
35	R	R
51	R	R
53	R	R

Figure S7. Herbicide responses of F7 progeny lines

(A) Florpyrauxifen-benzyl responses of bensulfuron-methyl-sensitive and -resistant lines. (B) Summary of sensitivity to florpyrauxifen-benzyl and bensulfuron-methyl in F7 progeny lines.

References

(1) Iwakami, S.; Endo, M.; Saika, H.; Okuno, J.; Nakamura, N.; Yokoyama, M.; Watanabe, H.; Toki, S.; Uchino, A.; Inamura, T. Cytochrome P450 CYP81A12 and CYP81A21 Are Associated with Resistance to Two Acetolactate Synthase Inhibitors in *Echinochloa Phyllopogon*. *Plant Physiol.* **2014**, *165* (2), 618–629.

(2) Katoh, K.; Standley, D. M. MAFFT Multiple Sequence Alignment Software Version 7: Improvements in Performance and Usability. *Mol. Biol. Evol.* **2013**, *30* (4), 772–780.

(3) Suyama, M.; Torrents, D.; Bork, P. PAL2NAL: Robust Conversion of Protein Sequence Alignments into the Corresponding Codon Alignments. *Nucleic Acids Res.* **2006**, *34* (Web Server issue), W609-12.

(4) Nguyen, L. T.; Schmidt, H. A.; Von, H. A.; Minh, B. Q. IQ-TREE: A Fast and Effective Stochastic Algorithm for Estimating Maximum-Likelihood Phylogenies. *Molecular biology and evolution* **2015**, *32* (1), 268–274.
